# Re-evaluation of the criteria for asymmetric amplatzer occluders in the closure of perimembranous ventricular septal defects: A case series report

**DOI:** 10.1097/MD.0000000000021356

**Published:** 2020-08-21

**Authors:** Gang Li, Hongyu Liao, Jinlin Wu, Kaiyu Zhou, Yimin Hua, Chuan Wang, Hongyu Duan, Xiaoqing Shi, Gang Wu, Yifei Li

**Affiliations:** a Key Laboratory of Birth Defects and Related Diseases of Women and Children of MOE, Department of Pediatrics, West China Second University Hospital, Sichuan University, Chengdu, Sichuan; b Department of Pediatrics, The Affiliated Hospital of Southwest Medical University, No. 25 Taiping Street, Luzhou, Sichuan, China.

**Keywords:** application criteria, asymmetric amplatzer occluder, receiver operating characteristic curve, ventricular septal defect

## Abstract

**Rationale:**

To discuss suitable criteria for the application of asymmetric Amplatzer occluders for perimembranous ventricular septal defects (pmVSDs).

**Patients concerns and diagnoses:**

We retrospectively studied 18 children with perimembranous VSDs who underwent attempted asymmetric occluder closure between January 2015 and December 2018 in our center.

**Interventions:**

Asymmetric Amplatzer occluders were attempted to be placed to all the enrolled patients. We analyzed the diameter of the defects with the receiver operating characteristic curve (ROC) values, the size of the occluders attempted, the presence of aneurysm and the presence of aortic valve prolapse for each patient. Then, for patients who experienced successful device implantation, the therapeutic efficiency was evaluated by follow-up.

**Outcomes:**

Only 5 out of a total of 18 patients completed successful device implantation. Compared with failed cases, successful cases demonstrated a significantly smaller VSD size (5.46 ± 1.03 mm vs. 8.73 ± 2.33 mm, *P* = 0.012) and had a low ratio of aortic valvar prolapse (20% vs. 76.92%, *P* = 0.026). Four out of 5 successful cases involved arrhythmia complications, but the rhythm of the heart recovered after drug treatment. According to the ROC and Youden analyses, the cut-off value of the defect size for successful asymmetric Amplatzer occluder implantation was no larger than 5.7 mm.

**Lessons:**

The application of an asymmetric Amplatzer occluder expands the range of indications for patients with superior localized VSD but is largely limited in cases with aortic valvar prolapse and large VSD sizes. All successful cases recovered from arrhythmia postprocedure.

## 1 Introduction

Transcatheter closure of perimembranous ventricular septal defects (VSDs) has been employed for almost 3 decades,^[1]^ and a series of studies has proven that it could be an alternative to surgical repair.^[2]^ However, several limitations still exist and prohibit this method from being the first choice for all kinds of VSD repair.^[3–5]^ For some specific localized VSDs, a symmetric occluder could not be implanted without an adverse impact on aortic valvar movement. The asymmetric device could be an alternative.^[6,7]^ However, few investigations have reported the outcomes of this type of occluder and how to choose it. When applying an asymmetric occluder in our center, we doubted the criteria for asymmetric Amplatzer occluders, and these criteria should be reconsidered. Herein, we reported our experience in a cohort who underwent attempted asymmetric occluder implantation and re-evaluated the criteria for the application of this device.

## 2 Methods

### 2.1 Patient population

This is a single center retrospective consecutive case series to analyze the optimal criteria for asymmetric Amplatzer occluder application in pmVSD patients. A cohort of 18 patients underwent attempted asymmetric occluder closure of perimembranous VSD with a SHAMA device between January 2015 and December 2018 in the Pediatric Heart Center of West China Second University Hospital, Sichuan University. This research was approved by the ethics committee of West China Second University Hospital, Sichuan University. Written consent forms were obtained from the parents of all participants. The distance of the lesion to the aortic valve rim was measured in the long-axis parasternal and apical 5-chamber views. The distance to the tricuspid valve rim was measured in the parasternal short-axis view. In addition, chest radiography and an electrocardiogram (ECG) were performed to identify pulmonary and cardiovascular concerns. Left ventricular angiography was used to confirm the shape, size, and location of the defect, as well as its distance to the valves. The research had been registry in our clinical research center, Sichuan University (2014-034).

The inclusion criteria were as follows: all patients were diagnosed with pmVSD by transthoracic echocardiography and cardiac angiography; closure of the defect using systematic Amplatzer occluders failed initially and was followed by subsequent attempts to use an asymmetric Amplatzer device (AGA Medical, Golden Valley, Minnesota, United States of America); and all patients met the basic inclusion criteria for transcatheter closure of congenital heart disease. The exclusion criteria were as follows: other types of cardiac malformation had been found, or some types of correction surgery had been previously completed; aim of the procedure was to close a residual shunt; several incidences of pulmonary hypertension (mean pulmonary artery pressure ≥70 mm Hg) had been identified; the aortic valves demonstrated a medium to severe prolapse that had been recorded as covering >1/3 size of the defect; severe arrhythmia had been diagnosed preintervention; and New York Hear Association (NYHA) functional class IV was identified.

### 2.2 Procedure

The pmVSD closure was performed under general anesthesia in children <10 years old and local anesthesia in older children. Dynamic ECG monitoring was applied during and after the procedure for 3 days. Right and left cardiac catheterization was performed via the percutaneous transfemoral route. The hemodynamics of the pulmonary vessels were measured, and pulmonary vessel resistance was evaluated before the final occlusion. The size of the VSD and the distance to the aortic and tricuspid valves were further confirmed by angiography. Wherever a cusp of the aortic valve and defect were superimposed, it was considered a contraindication for transcatheter closure. The size of the device was usually selected to be 1 to 2 mm larger than the defect measured by angiocardiography. Heparin (100 unit/kg) was administered to all patients after successful femoral artery access. The transcatheter closure of the pmVSD was performed following the standard procedure, as described previously. ECG was performed on the first, third, and seventh days after the procedure. Aspirin was taken for 6 months after the procedure.^[8]^

### 2.3 Follow-up and classification of complications

All patients who received an asymmetric Amplatzer occluder underwent follow-up examinations at 1, 3, and 6 months. During follow-up, clinical characteristics, an ECG and echocardiography were performed. Arrhythmias were monitored by 12-lead ECG and Holter monitor. In the case of unexpected complications, patients underwent chest x-rays to demonstrate the position and shape of the occluder. Of note, arrhythmias detected within 24 hours after the procedure were not considered complications. As there were a limited number of patients enrolled for this study, we ensured that every patient would complete the routine follow-up hospital visit procedures by providing reminders by telephone, and we allowed a 3 to 5 days extension of the scheduled time point.

### 2.4 Statistical analysis

Continuous variables were expressed as the mean ± standard deviation (SD). Differences between 2 groups were analyzed by independent *t* tests. Noncontinuous variables were expressed as proportions, and differences between groups were analyzed by chi-squared test. The receiver operating characteristic curve (ROC) was used to analyze the predictive value of potential factors for successful implantation of asymmetric occluders by SPSS software version 20.0 (SPSS, Inc., Chicago, IL). Then, the Youden index was calculated by MEDCALC software version 19.0 (MedCalc Software Ltd., Ostend, Belgium) to identify the optimal cut-off value for the ROC.

## 3 Results

Among all the patients, there were 8 women and 10 men, and the average age was 32.16 ± 11.68 months, while the average weight was 12.58 ± 2.36 kg. All patients were diagnosed with superior localized perimembranous VSDs with no recorded distance between the upper border of the defect and the aortic valves. The size of the defects was 7.82 ± 2.52 mm in this cohort, and the size of the asymmetric Amplatzer occulders that were attempted to be implanted was 10.29 ± 2.47. Perimembranous aneurysms were detected in 14 children, while aortic valve prolapses were recorded in 11 cases. The average duration of the procedure taken was 50.72 ± 9.38 mm. Every patient received an evaluation before anesthesia by an interventional cardiologist, cardiac surgeon, and echocardiographic physician, who collectively decided to attempt implantation of an asymmetric occluder.

**Figure 1:**
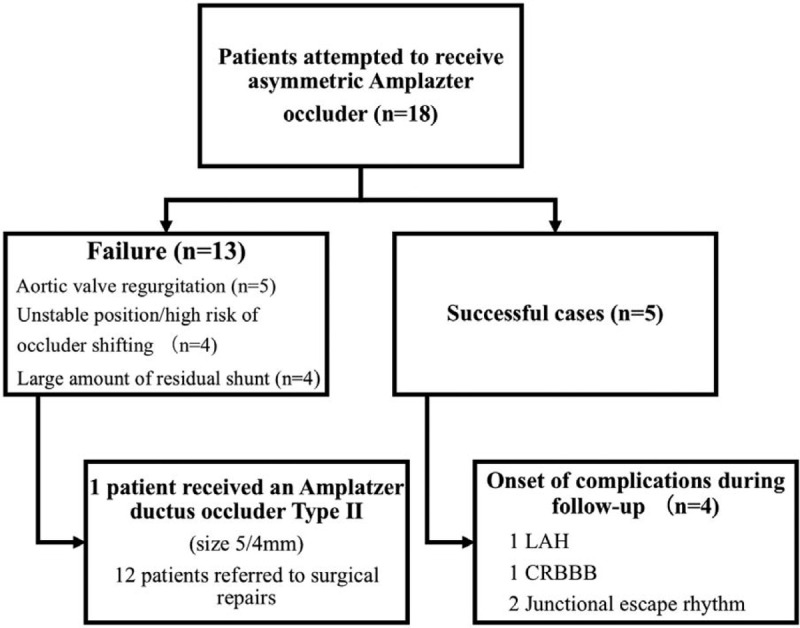
The flow chart of all the attempted cases for asymmetric Amplatzer occluder.

As shown in Table 1, only 5 out of 18 children achieved successful device implantations. Among the 13 failed cases, 5 cases demonstrated unacceptable aortic valvar regurgitation, 4 cases demonstrated unstable device position or a high risk of occluder shifting, and 4 cases maintained a large residual shunt. Twelve of them were referred for surgical repair, and 1 small VSD received an Amplatzer Ductus Occluder II device instead (Fig. 1). There was no significant difference in the evaluation of age, weight, or presence of aneurysms between successful and failed cases (Table 1). However, the successful case demonstrated a significantly smaller VSD size and device choice (Table 1). Interestingly, only 1 (20%) patient had mild aortic valvar prolapse among the successful cases, but 10 (76.92%) patients suffered mild to medium aortic valvar prolapse.

**Table 1:**
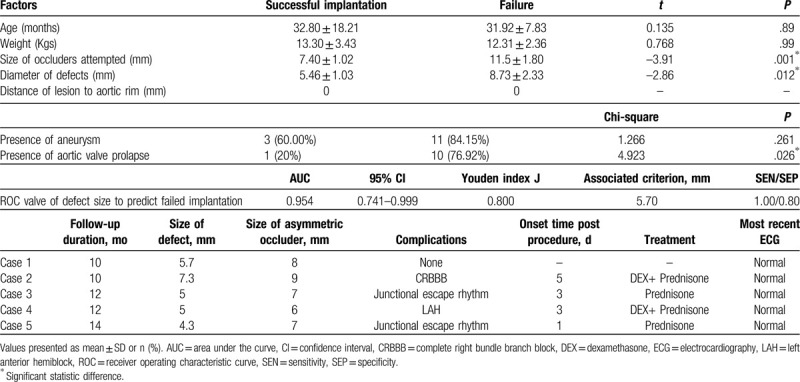
The analysis of potential risk factors for successful implantation of asymmetric occluder and outcomes.

The size of the defect was considered to be a predictive factor for successful implantation. ROCs were drawn with an area under the curve (AUC) of 0.954 (95% CI 0.741–0.999). Then, Youden analysis revealed its J index as 0.800 and provided a cut-off valve of 5.70 mm (sensitivity, 1.00; specificity, 0.80).

All patients were followed up for at least 10 months (range 10–14 months). Four out of 5 successful cases suffered arrhythmia complications. As shown in Table 1, 3 types of arrhythmias occurred between 1 and 5 days postprocedure, including complete right bundle branch block (CRBBB), left anterior hemiblock (LAH), and junctional escape rhythm. After the administration of dexamethasone (1 mg/kg for 3 days) and prednisone (1 mg/kg for 3–5 days), the rhythm of the heart recovered and was maintained to recent observations.

## 4 Discussion

The asymmetric Amplatzer occluder was designed to solve the problem of superior localized VSD dealing with the dilemma of aortic valvar prolapse.^[9]^ According to previous opinion, the asymmetric Amplatzer occluder is invented to expanded the indication for transcatheter closure of pmVSD.^[10,11]^ So that the asymmetric occluder has been used as an alternation for the failure closure of systematic Amplatzer occluder. Due to the specific structure characteristics of pmVSD, failure of device closure is usually considered with the causes of large defect, aortic valvar prolapse, and severe complications. Based on this point, several previous researches reported the attempt of closure for large superior localized VSD using asymmetric Amplatzer occluder.^[12,13]^ However, according to our analysis, the asymmetric device is definitely an optimal choice for superior perimembranous VSD, but it is largely limited in cases with aortic valvar prolapse. Due to the unique structure of asymmetric devices, they would not be a good choice for large defects due to a very high failure rate. Based on the ROC and Youden analyses, the cut-off value of the defect size is 5.70 mm, indicating that the criteria for asymmetric occluder implantation should be characterized as superior localized perimembranous VSDs that are smaller than 6-mm without or with mild aortic valvar prolapse. So that, the aim of this study is to re-evaluation the indication of asymmetric Amplatzer occluder. And the results demonstrated the application of such device is not simply an alternation or supplementary for the failure closure using regular device. The asymmetric Amplatzer occulder revealed a very limited indication for superior localized pmVSD with small defect size, trying to avoid the adverse impacts on aortic valvar.

Besides, the superior location of VSDs is supposed to have a lower rate of arrhythmia. In this case cohort, most of the successful cases suffered short-term arrhythmia postprocedure. This is considered due to the longer duration of the procedure and the repeated routine wire establishment through the defects, and all the arrhythmias were benign and disappeared after timely treatments. In addition, the asymmetric Amplatzer occluder often puts the long side down to avoid the aortic valve or tricuspid valve, making it easier to compress the conduction bundle.^[8,14]^ Therefore, arrhythmias should not be taken as a limitation for asymmetric Amplatzer occluder implantation. To address this issue, we consider that the longer time of the interventional procedure and the delivery sheath passing through the septum are the reasons for the occurrence of arrhythmias.

### 4.1 Study limitations

Despite this is one of the largest series to describe the indication criteria for asymmetric Amplatzer occluders for pmVSD. However, the application of the result is still limited by the few cases enrolled in the research. And it also requires an additional large, prospective, multi-center, and well-designed study to contribute to better understanding the most optimal indication for asymmetric occluder.

## 5 Conclusion

In summary, a VSD patient should meet the following criteria to receive an asymmetric Amplatzer occluder device: have a perimembranous VSD that meets the criteria for transcatheter closure of VSD; have a medium defect size <6 mm; only experiencing mild or no aortic valvar regurgitation; and the presence of aneurysm does not influence the success rate of implantation.

## Author contributions

**Conceptualization:** Kaiyu Zhou, Yimin Hua, Yifei Li.

**Data curation:** Hongyu Liao, Kaiyu Zhou, Yifei Li.

**Formal analysis:** Kaiyu Zhou, Yifei Li.

**Funding acquisition:** Kaiyu Zhou, Yimin Hua, Yifei Li.

**Investigation:** Hongyu Liao, Yimin Hua, Yifei Li, Gang Li.

**Methodology:** Hongyu Liao, Yimin Hua, Chuan Wang, Xiaoqing Shi.

**Project administration:** Yimin Hua.

**Resources:** Chuan Wang, Hongyu Duan.

**Software:** Chuan Wang, Hongyu Duan, Xiaoqing Shi, Gang Li.

**Supervision:** Yifei Li.

**Validation:** Chuan Wang, Xiaoqing Shi, Yifei Li.

**Writing – original draft:** Hongyu Liao, Yifei Li.

**Writing – review & editing:** Yifei Li, Gang Li.
